# The complete chloroplast genome sequence of *Viburnum Japonicum* (Adoxaceae), an evergreen broad-leaved shrub

**DOI:** 10.1080/23802359.2018.1462121

**Published:** 2018-04-12

**Authors:** Won-Bum Cho, Eun-Kyeong Han, Hyeok Jae Choi, Jung-Hyun Lee

**Affiliations:** a Department of Biology Education, Chonnam National University, Gwangju, Republic of Korea;; b Department of Biology and Chemistry, Changwon National University, Changwon, Gyeongnam, Republic of Korea

**Keywords:** Chloroplast genome, evergreen shrub, phylogenetic analysis, *Viburnum japonicum*

## Abstract

The wax-leafed *Viburnum japonicum* (Adoxaceae) is an evergreen shrub distributed in Japan, Korea, and Taiwan. We sequenced its complete chloroplast (cp) genome to examine its phylogenetic relationship within Dipsacales. This genome is 158,614 bp long and features a large single-copy region (87,059 bp) and a small single-copy region (18,523 bp), separated by two inverted-repeat regions (26,516 bp each). It contains 128 genes, including 84 coding genes, eight rRNAs, and 36 tRNAs. The overall GC content is 38.1%. Our phylogenetic tree showed that *V*. *japonicum* is closely related to *V*. *utile* and is clustered together with four species in the family Adoxaceae.

Within Dipsacales, the *Viburnum* genus has traditionally been regarded as a group within Caprifoliaceae along with *Sambucus* and *Sinadoxa* (Cronquist 1988). The latter two also belong to Adoxaceae, under the classification of APG (Donoghue et al. 2001). Analyses of molecular and morphological data suggest that *Viburnum* is the basal-most lineage in the family Adoxaceae (Jacobs et al. 2010). Therefore, understanding the evolutionary relationships of *Viburnum*, a major basal group, would provide a powerful phylogenetic utility and, in particular, convincing evidence for early-diverging Adoxaceae. *Viburnum japonicum* (Thunb.) Spreng. is an evergreen shrub distributed in the subtropical regions of Korea, Japan, and Taiwan (Yang and Chiu 1998; Hong and Im 2003). Here, we report its complete chloroplast (cp) genome sequence.

We collected samples of *V*. *japonicum* from Is. Gageo-do, Jeollanam-do Province, Korea (N 34° 03′ 59″, E 125° 07′ 04″). The voucher specimen was stored at the herbarium in the Department of Biology Education, Chonnam National University (BEC: Lee. 16101504). Total genomic DNA was extracted with a DNeasy Plant Mini Kit (Qiagen, Seoul, Korea), and pair-end sequencing (300 × 300 bp) was performed using the Illumina Miseq platform (LAS, Seoul, Korea). This presented 13,920,280 raw reads. After trimming the sequences, clean reads were mapped with the reference cp genome for *V*. *utile* (NC_032296), using Geneious 10.2.3 (Kearse et al. 2012). On that genome, 93,629 reads were assembled with an average of 177X coverage (max: 286X, min: 60X). The annotation was separately performed using DOGMA (Wyman et al. 2004) and tRNAscan-SE (Schattner et al. 2005). The annotated cp genome sequence was then deposited in GenBank with Accession Number MH036493. To construct the phylogenetic tree, we performed maximum likelihood (ML) analysis with RAxML v.8.0 (Stamatakis 2014), using complete cpDNAs of nine related species from the NCBI database aligned with MAFFT (Katoh and Toh 2010).

The cp genome of *V*. *japonicum* is 158,614 bp long, with 38.1% GC content. It has two inverted-repeat (IR) regions of 26,516 bp that separate a large single-copy region (87,059 bp) and a small single-copy region (18,523 bp). The genome contains 128 genes: 84 protein-coding genes, eight rRNA genes, and 36 tRNA genes. Seventeen genes are duplicated in the IR region. The ML phylogenetic tree, with 1000 bootstrap replications, is monophyletic with two independent clades in Dipsacales. *Viburnum japonicum* is closely related to *V*. *utile* and clusters together with four species in the family Adoxaceae (Figure 1). The chloroplast genome of *V*. *japonicum* provides information for further phylogenetic studies and genome resources within Dipsacales.

**Figure 1.**
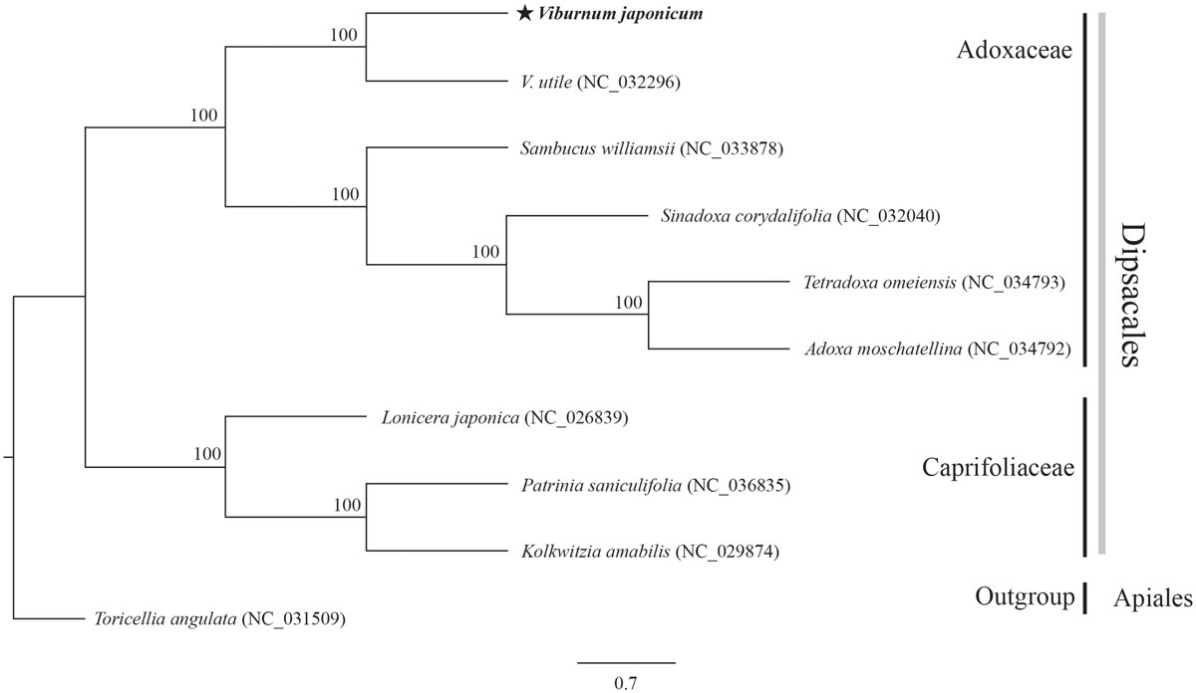
Maximum-likelihood phylogenetic tree based on complete chloroplast genomes of 10 species, including *Toricellia angulata* as outgroup. Numbers above nodes indicate bootstrap values with 1000 replicates.
